# The global prevalence of low back pain in pregnancy: a comprehensive systematic review and meta-analysis

**DOI:** 10.1186/s12884-023-06151-x

**Published:** 2023-12-02

**Authors:** Nader Salari, Aida Mohammadi, Mahvan Hemmati, Razie Hasheminezhad, Salim Kani, Shamarina Shohaimi, Masoud Mohammadi

**Affiliations:** 1 Department of Biostatistics, School of Health, Kermanshah University of Medical Sciences, Kermanshah, Iran; 2 Sleep Disorders Research Center, Kermanshah University of Medical Sciences, Kermanshah, Iran; 3 Student Research Committee, Kermanshah University of Medical Sciences, Kermanshah, Iran; 4 Department of Orthopedic Surgery, School of Medicine, Shahid Beheshti University of Medical Sciences, Tehran, Iran; 5 Department of Biology, Faculty of Science, University Putra Malaysia, Serdang, Selangor Malaysia; 6 Cellular and Molecular Research Center, Gerash University of Medical Sciences, Gerash, Iran

**Keywords:** Low back pain, Pregnancy, Systematic review, Meta-analysis

## Abstract

**Background:**

Back pain during pregnancy is often considered as an unavoidable problem and can reduce the quality of life or disability of pregnant women. The aim of this study is to determine the global prevalence of back pain in pregnancy based on a systematic review and meta-analysis.

**Methods:**

In this study, Researchers systematically searched electronic databases PubMed, Scopus, Web of Science, Embase, ScienceDirect, and Google Scholar search engines for studies until September 2023. To analyze data, the random effects model was used, and the heterogeneity of the studies was checked with the I2 index. Data analysis was performed by software (Version 2 Comprehensive Meta-Analysis).

**Results:**

In the review of 28 studies with a sample size of 12,908 people, the I^2^ heterogeneity test showed high heterogeneity (I^2^: 98.4). Based on this, the random effects method was used to analyze the results. Therefore, the meta-analysis reported the global prevalence of back pain at 40.5 (95% CI: 33–48.4) during pregnancy. Also, according to the meta-analysis, the global prevalence of back pain in the first trimester of pregnancy is 28.3 (95%CI: 10.5–57.1), in the second trimester is 36.8 (95%CI: 30.4–43.7) and in the third trimester of pregnancy was reported as 47.8 (95% CI: 37.2–58.6).

**Conclusion:**

In this meta-analysis, the overall prevalence of back pain in pregnant women was reported to be significant, so it is necessary for health policymakers to pay more attention to complications during pregnancy, in addition to increasing society's awareness of pregnant mothers, with timely diagnosis and treatment of such disorders, it can lead to improvement; and reduction in Complications caused by pregnancy and becoming more pleasant during pregnancy.

## Background

Pregnancy back pain refers to a type of back pain that appears during pregnancy, and the person has no history of back pain before that [1]. Pregnancy back pain is one of the most common musculoskeletal pains that most women experience for the first-time during pregnancy and may cause many problems and disabilities for them [2].

Almost 70% of pregnant women suffer back pain during pregnancy, and in many of them, the severity of back pain prevents them from doing daily activities and leads to rest [2]. These back pains are more common in the second trimester of pregnancy, but in some cases, pregnancy backaches may occur from the first trimester [3]. Back pain usually starts between the fifth and seventh months of pregnancy, and back pain related to pregnancy may continue up to three months after delivery [4]. Also, women who had back pain before pregnancy are twice as likely to suffer from this condition; the frequency of back pain increases with the age of the person at the time of pregnancy and the number of pregnancies [5].

As mentioned, one of the conditions that make women prone to back pain is pregnancy. Pregnancy causes a change in a person's physical state, decreases the ability to bear weight and heavy loads, and increases complaints of muscle and skeletal pains [6]. During pregnancy, the mother's weight increases by an average of 11 to 12 kg, and the hormonal and biomechanical changes in the mother's body make her susceptible to a variety of musculoskeletal problems, such as back pain, pelvic pain, sciatica pain, coccyx pain, carpal tunnel syndrome, and Restless legs syndrome (RLS) [7]. Among the other factors that have been mentioned as factors affecting back pain during pregnancy are the history of back pain in a previous pregnancy or any previous history of back pain, the young age of the mother, repeated childbirths, stress, physical pressures at work, and history. Trauma pointed to the back or pelvis [8]. Also, in the last months of pregnancy, with the increase in the weight of the fetus, the pressure on the spinal nerves increases, which in turn causes the back pain to intensify; In addition, endocrine changes such as the increase of relaxin and progesterone hormones are effective in the occurrence of back pain during pregnancy [9].

Most pregnant women who suffer from back pain consider this a part of the pregnancy process, which causes them not to take any special measures to solve it [9]. But this disorder can cause disability, reduce the quality of life, or disable pregnant women [10]. Also, not treating pregnancy back pain in the not-too-distant future can lead to the need for surgery, and after pregnancy can also, various events in the life of mothers leave adverse effects [11] and lead to the recurrence of this condition in subsequent pregnancies, therefore, the treatment measures of these mothers should be taken into consideration [12].

The treatment of back pain in pregnancy depends on the stage of pregnancy, underlying causes, aggravating factors, and the presence of other medical conditions [11, 12]. The management approach typically includes treatments from an obstetrician, orthopedic specialist, neurologist, and/or neurosurgeon [7–12]. Maintaining an optimal level of function throughout the gestation period and having the least amount of discomfort are the main goals of treatment for back pain during pregnancy [7–12]. Treatment and management options may include Postural correction, supported side-sleeping, lumbar roll while sitting, limiting standing and walking, and antenatal exercises. also, Healthy pregnant women can exercise for at least 150 min per week or 20–30 min of moderate to intense aerobic activity [7–12].

Due to the high rate of back pain during pregnancy, we decided to review the studies conducted in this field to do a general statistical survey on the global prevalence of back pain during pregnancy. The purpose of this study is a systematic review and meta-analysis of the global prevalence of back pain during pregnancy, which can be considered critical evidence to pay attention to the issue of back pain during pregnancy and its complications in pregnant mothers around the world.

## Method

In this systematic review and meta-analysis, the primary search was conducted until September 2023. to find relevant studies in 5 databases PubMed, Web of Science, Google Scholar, Scopus, ScienceDirect, and Embase, using the keywords Prevalence, outbreak, Burden, pregnancy, gravidity, conception, gestation, "back pain," "low back pain" were searched. To maintain the comprehensiveness of the search, Researchers applied no restrictions on the year of publication of the articles, and the identified information was transferred to the information management software (Endnote). The list of references used in the identified related articles was reviewed manually to maximize the number of relevant studies. The searches were last updated in late September 2023.

### Inclusion and exclusion criteria

Study inclusion criteria:

1. Cross-sectional studies that reported the prevalence of back pain in pregnancy
2. Studies whose full text was available.
3. Studies that provided sufficient data (sample size, prevalence)
4. Studies were in English

Study exclusion criteria:

1. Case report and case series studies
2. Review studies
3. Repetitive studie
4. Studies with insufficient data (lack of information about the prevalence and number of samples)
5. Studies that were not in English.
6. Letter to the editor, articles presented in conferences, secondary studies, theses

### Study selection

The selection of studies was made according to PRISMA guidelines. First, the studies duplicated in different databases were excluded from this study. Then Researchers made the initial selection and review of the articles according to the titles and abstracts, and irrelevant articles were removed based on the inclusion and exclusion criteria. Then we evaluated their full text based on inclusion and exclusion criteria, and irrelevant studies were removed at this stage. To avoid bias, all the steps of reviewing sources and extracting data were done by two researchers independently. In cases where there was a difference of opinion between two researchers, the article was reviewed by a third person.

### Quality evaluation

A checklist was used according to observational studies, to validate and evaluate the quality of articles. The Strengthening the Reporting of Observational Studies in Epidemiology checklist (STROBE) consists of six scales. Based on this, articles with a score of 16 and above were considered good methodological quality. Essays with scores below 16 were supposed to be of poor methodological quality and therefore excluded from the study.

### Data extraction

Data extraction was done by two researchers using a previously prepared checklist. This checklist included: the first author's name, year of publication, study location, sample size, category and the average age of women, prevalence of back pain in pregnancy, and study tools.

### Statistical analysis

Researchers entered the results extracted from this study into the software Version 2 (Comprehensive Meta-Analysis), and the heterogeneity of the studies was used through the I^2^ test. Also, to check the publication bias, the Funnel plot and the Egger test were used at a significance level of 0.05.

## Results

**Fig. 1:**
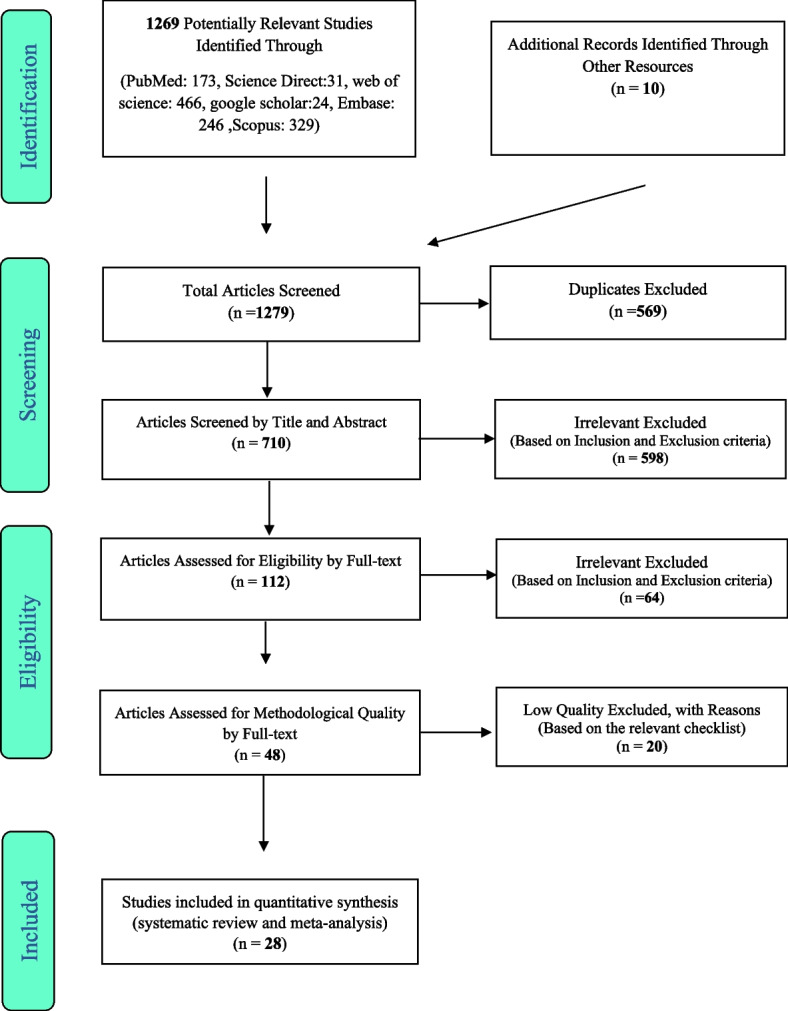
The flowchart on the stages of including the studies in the systematic review and meta-analysis (PRISMA 2009)

In this systematic review and meta-analysis of studies, the global prevalence of back pain in pregnancy has been shown, which was systematically evaluated based on the PRISMA guidelines. One thousand two hundred sixty-nine articles were searched through databases, and ten related articles were identified through manual search and transferred to the information management software (Endnote). Five hundred sixty-nine articles were removed due to duplication. In the screening stage, the title and abstract of the studies were evaluated, and 598 articles were excluded based on the inclusion and exclusion criteria. In the merit evaluation phase, 64 articles were excluded through the full-text study based on the inclusion and exclusion criteria. In the qualitative evaluation phase, the studies with poor methodological quality were excluded by analyzing the full text of the articles and based on the score obtained from the STROBE checklist. Finally, 28 studies were included in the final evaluation. The information from these 28 studies is reported in Fig. 1 and Table 1.

**Table 1:** Prevalence of back pain in pregnancy

Author	Year	Reign	Continent	25 ± 4	Type of study	pregnant sample size	Number of pregnant with low back pain	Prevalence of low back pain	Instrument
Shijagurumayum Acharya et al. [13]	2019	Nepal	Asia	27.51 ± 5.34	cross-sectional study	1284	436	34	self-reported questionnaires
Ghaderi et al. [14]	2012	Iran	Asia	27.8 ± 5	cross-sectional study	235	121	58.2	questionnaire-Quebec Back Pain Disability Scale
Hollisaz et al. [15]	2013	Iran	Asia	31 ± 7.8	cross-sectional study	230	146	63.5	questionnaire-Quebec Back Pain Disability Scale
Sedighi et al. [16]	2007	Iran	Asia	28.98 ± 5.02	cross-sectional study	252	71	28.4	interview
Rabiee et al. [12]	2018	Iran	Asia	25.83 ± 5.91	cross-sectional study	514	355	69.06	VAS Scale
Manyozo et al. [8]	2019	Malawi	Africa	22.7 ± 4.5	cross-sectional study	404	249	62	researcher administered questionnaire
Skaggs et al. [17]	2007	USA	America	25.98 ± 5.08	cross-sectional study	401	153	38.15	self-reported questionnaires
Mohseni-Bandpei et al. [18]	2009	Iran	Asia	27.1 ± 5.4	cross-sectional study	1062	427	40.2	self-reported questionnaires
Ayanniyi et al. [19]	2006	Nigeria	Africa	< 35	cross-sectional study	2187	669	34.9	researcher administered questionnaire
Pierce et al. [20]	2012	Australia	Australia	27.14 ± 5.46	cross-sectional study	64	-	33	researcher administered questionnaire
Ansari et al. [21]	2010	Iran	Asia	29.88 ± 4.80	cross-sectional study	103	59	57.3	self-report
Mogren et al. [22]	2005	Sweden	Europe	29.05 ± 5.2	cross-sectional study	891	634	71.7	self-report
Al-Sayegh et al. [23]	2012	Kuwait	Asia	25.4 ± 4	cross-sectional study	255	35	13.8	researcher administered questionnaire
Mousavi et al. [24]	2007	Iran	Asia	29.85 ± 3.84	cross-sectional study	325	91	28	survey questionnaire
Starzec et al. [25]	2019	Poland	Europe	23.80 ± 3.20	cross-sectional study	189	32	17	interview
Gupta et al. [26]	2014	India	Asia	28.85 ± 3.87	cross-sectional study	227	68	29.9	researcher administered questionnaire
Rodrigues et al. [27]	2011	Brazil	America	31.5 ± 4.8	cross-sectional study	66	49	75	self-report
Wang et al	2004	USA	America	30.1 ± 4.8	cross-sectional study	950	42	6.5	self-report
Malmqvist et al. [28]	2012	Norway	Europe	35–54	cross-sectional study	569	124	50	researcher administered questionnaire
Stapleton et al. [9]	2002	Australia	Australia	24.24 ± 3.20	cross-sectional study	397	269	68	questionnaire on demographic
Saxena et al. [7]	2019	India	Asia	27.1 ± 9.9	cross-sectional study	200	162	80	researcher administered questionnaire
Mazicioglu et al. [29]	2006	Turkey	Asia	32.8 ± 4.4	cross-sectional study	1600	734	54.1	self-report
Tavares et al. [4]	2019	Toronto	America	27.09 ± 5.66	cross-sectional study	343	80	26.3	self-report
Shafi et al. [30]	2021	Pakistan	Asia	25 ± 4	cross-sectional study	160	55	68.8	Quebec, Oswestry back pain disability indexes

In the studies included in Table 2, the highest prevalence of back pain in the first trimester of pregnancy is related to Rabei et al. in Iran in 2018, 63.3% [12], and the lowest prevalence is associated with the study by Weis et al. in Toronto, Canada in 2020 was with 5.9% [31].

**Table 2:** Prevalence of back pain in the first trimester of pregnancy

Author	Year	Reigen	Continent	Age	Type of study	pregnant sample size	Number of pregnants with back pain	Prevalence of back pain	Instrument
Rabiee et al. [12]	2018	Iran	Asia	28.98 ± 5.02	cross-sectional study	98	62	63.3	VAS Scale
Sencan et al. [32]	2018	Turkey	Asia	26.5 ± 5.5	cross-sectional study	1500	251	17.43	self-report
Weis et al. [31]	2018	Toronto	America	28.4 ± 8.4	cross-sectional study	287	17	5.9	self-report
Nazari et al. [33]	2020	Iran	Asia	-	cross-sectional study	550	117	59.1	self-report

In the studies included in Table 3, the highest prevalence of back pain in the second trimester of pregnancy was related to Rabei et al. in 2018, 63.4% [12], and the lowest majority was related to the study by Shijagurumayum et al. in 2019 with 34% [13].

**Table 3:** Prevalence of back pain in the second trimester of pregnancy

Author	Year	Reigen	Continent	Age	Type of study	pregnant sample size	Number of pregnants with back pain	Prevalence of back pain	Instrument
Rabiee et al. [12]	2018	Iran	Asia	28.98 ± 5.02	cross-sectional study	145	92	63.4	VAS Scale
Sencan et al. [32]	2018	Turkey	Asia	26.5 ± 5.5	cross-sectional study	1500	561	37.4	self-report
Shijagurumayum et al. [13]	2019	Nepal	Asia	25 ± 4	cross-sectional study	1284	437	34	self-reported
Stapleton et al. [9]	2002	Australia	Australia	-	cross-sectional study	1120	397	35.5	researcher administered questionnaire
Weis et al. [31]	2018	Toronto	America	28.4 ± 8.4	cross-sectional study	287	109	38	self-report
Nazari et al. [33]	2020	Iran	Asia	-	cross-sectional study	550	110	62.85	self-report

In the studies in Table 4, the highest prevalence of back pain in the third trimester of pregnancy related to Rabei et al. in 2018 was 74.2% [34], and the lowest majority was related to the study by Tariq et al. in 2018 in Pakistan with 26.3% [35].

**Table 4:** Prevalence of back pain in the third trimester of pregnancy

Author	Year	Reigen	Continent	Age	Type of study	pregnant sample size	Number of pregnants with back pain	Prevalence of back pain	Instrument
Rabiee et al. [34]	2018	Iran	Asia	28.98 ± 5.02	cross-sectional study	271	201	74.2	VAS Scale
Kovacs et al. [36]	2012	spain	Europe	32.25 ± 1.46	cross-sectional study	1185	825	71.3	self-report
Robinson et al. [37]	2010	Norway	Europe	31.3 ± 4.20	cross-sectional study	283	-	52	VAS Scale
Sencan et al. [32]	2018	Turkey	Asia	26.5 ± 5.5	cross-sectional study	1500	688	45.86	self-report
Khan et al. [38]	2017	Pakistan	Asia	24.56	cross-sectional	96	66	68.8	researcher administered questionnaire
Tariq et al. [35]	2018	Pakistan	Asia	26.30 ± 4.5	cross-sectional study	1000	263	26.3	researcher administered questionnaire
Madadi-Shad et al. [39]	2018	Pakistan	Asia	-	cross-sectional study	560	227	40.6	self-report
Omoke et al. [40]	2021	Nigeria	Africa	29.33 ± 4.8	cross-sectional study	471	138	58.70	researcher administered questionnaire
Berber et al. [6]	2020	Turkey	Asia	28.09 ± 5.58	cross-sectional study	400	182	45.5	researcher administered questionnaire
Weis et al. [31]	2018	Toronto	America	28.4 ± 8.4	cross-sectional study	287	161	56.1	self-report
Nazari et al. [33]	2020	Iran	Asia	-	cross-sectional study	550	123	69.49	self-report

### The global prevalence of back pain during pregnancy

**Fig. 2:**
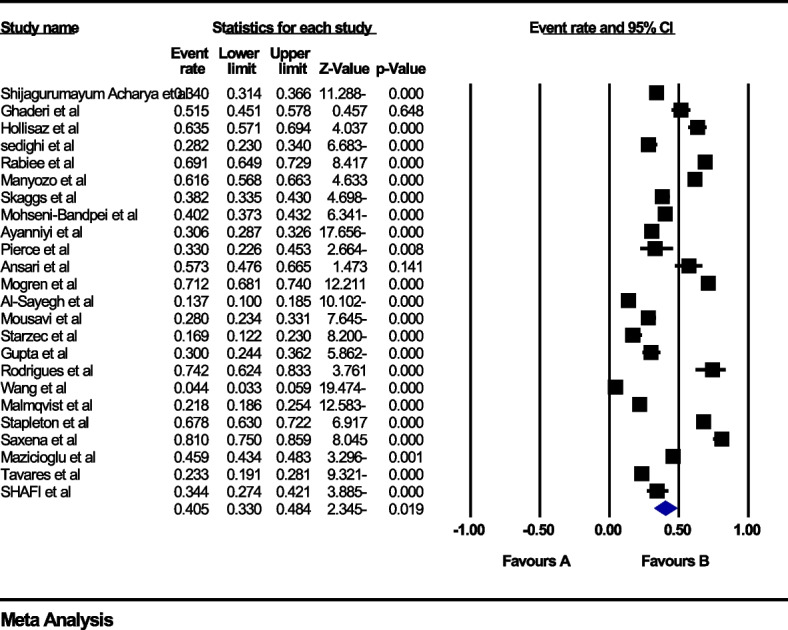
Forest plot of the global prevalence of back pain during pregnancy based on random effects method

**Fig. 3:**
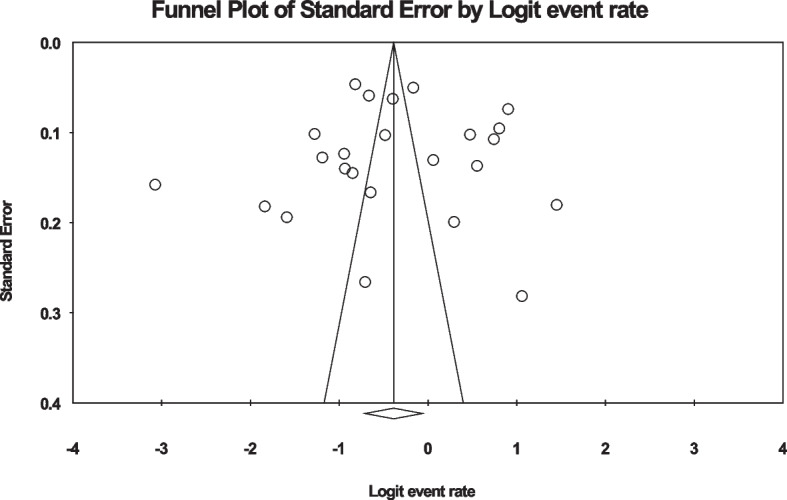
Funnel plot of the publication bias in the reviewed studies

In the review of 28 studies with a sample size of 12,908 people, the I^2^ heterogeneity test showed high heterogeneity (I^2^: 98.4) and based on this, we used the random effects method to analyze the results, therefore, based on the meta-analysis, the global prevalence of back pain in 40.5 (95%CI: 33–48.4) during pregnancy was reported (Fig. 2). Also, the study of diffusion bias in the studies through the Egger test shows the absence of publication bias in the analyses (p: 0.949) (Fig. 3).

### The global prevalence of back pain in the first trimester of pregnancy

**Fig. 4:**
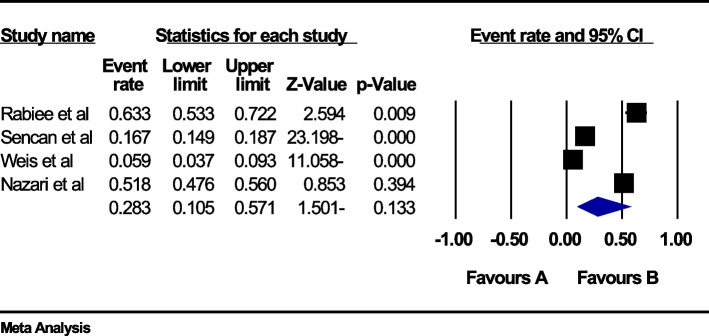
Forest plot of the global prevalence of back pain in the first three months of pregnancy based on the random effect's method

**Fig. 5:**
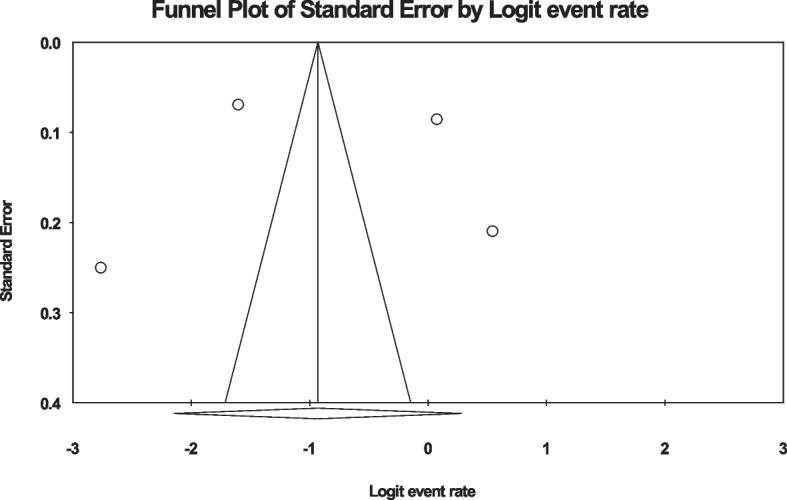
Funnel plot of the distribution bias in the reviewed studies

In the review of 4 studies with a sample size of 2435 people, the I^2^ heterogeneity test showed high heterogeneity (I^2^: 99.1). Based on this, we used the random effects method to analyze the results. Therefore, based on the meta-analysis, the global prevalence of back pain in the first three months of pregnancy was reported as 28.3 (95%CI: 10.5–57.1) (Fig. 4). The analysis of publication bias in the studies through the Egger test shows the absence of publication bias in the studies (p: 0.903) (Fig. 5).

### The global prevalence of back pain in the second trimester of pregnancy

**Fig. 6:**
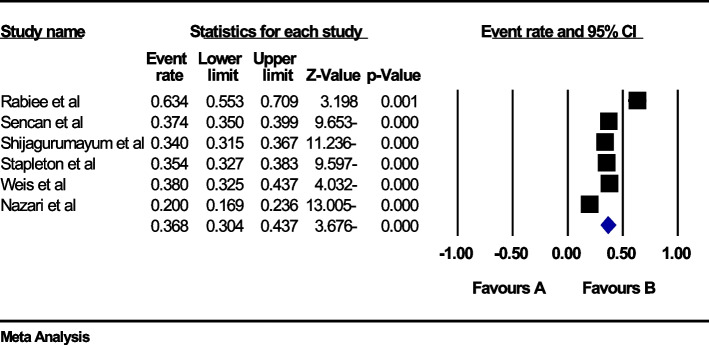
Forest plot of the global prevalence of back pain in the second trimester of pregnancy based on the random effect's method

**Fig. 7:**
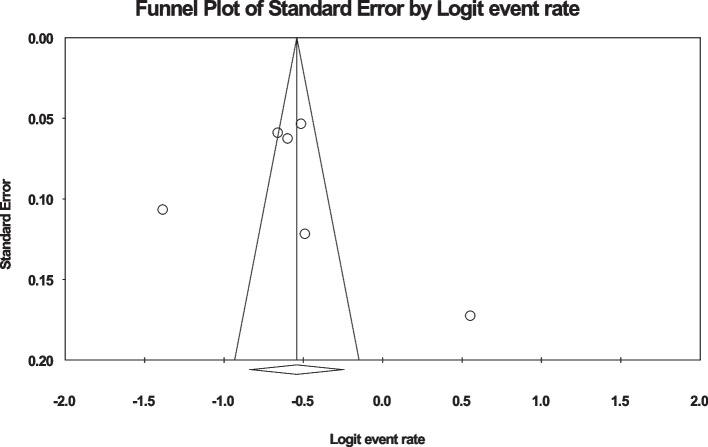
Funnel plot of the publication bias in the reviewed studies

In the review of 6 studies with a sample size of 4886 people, the I^2^ heterogeneity test showed high heterogeneity (I^2^: 95.1) and based on this, the random effects method was used to analyze the results, so based on the meta-analysis, the global prevalence of back pain in The second trimester of pregnancy was reported to be 36.8 (95%CI: 30.4–43.7) (Fig. 6). Also, the study of publication bias in the studies through the Egger test shows the absence of publication bias in the studies (p: 0.752) (Fig. 7).

### The global prevalence of back pain in the third trimester of pregnancy

**Fig. 8:**
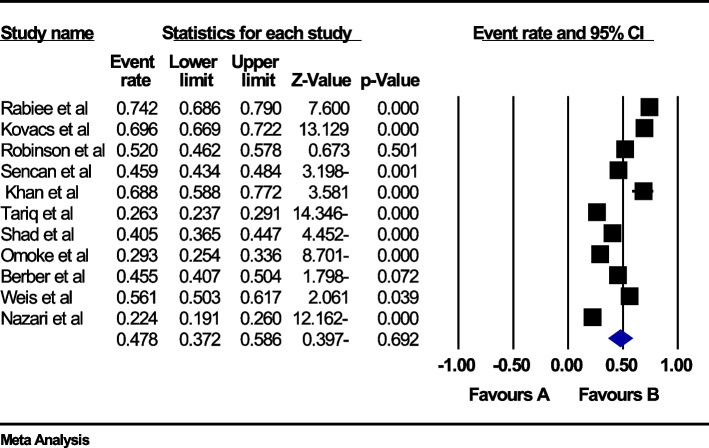
Forest plot of the global prevalence of back pain in the third trimester of pregnancy based on the random effect's method

**Fig. 9:**
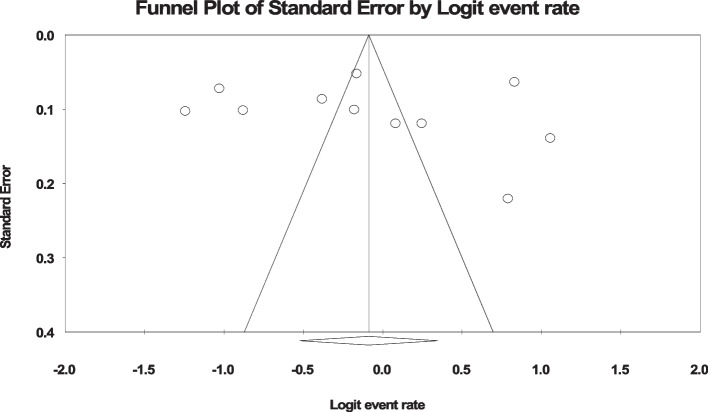
Funnel plot of the publication bias in the reviewed studies

In the review of 11 studies with a sample size of 6603 people, the I^2^ heterogeneity test showed high heterogeneity (I^2^: 98.5), and based on this, the random effects method was used to analyze the results, so based on the meta-analysis, the global prevalence of back pain in During pregnancy, 47.8 (95%CI: 37.2–58.6) was reported (Fig. 8), and the study of diffusion bias in the studies through the Egger test shows the absence of publication bias in the studies (p: 0.885) (Fig. 9).

## Discussion

The present study is a systematic review and meta-analysis of the global prevalence of back pain in pregnancy. According to the results of the present study, the overall prevalence of back pain in pregnant women was 40.5%.

Back pain is a multi-caused disease that primarily affects active people in society [41]. This disease is the most common cause of activity limitation in people under 45 years of age, the second most common reason for visiting a doctor, and the fifth most common reason for hospitalization, and it has a tremendous negative effect on the financial and economic situation of societies [42].

Back pain is a common symptom in pregnant women. During pregnancy, the body is affected by physiological and hormonal changes due to changes in the structure of the vertebral column and pressure on the nervous system, which causes back pain [4]. Among these changes is the laxity of the ligaments of the sacroiliac joints, lordosis, which causes the body's center of gravity to shift forward, inactivity, fatigue, and stretching of the muscles of the lumbar region, which increases the pressure parallel to the lumbar vertebral ligaments and causes back pain during pregnancy [13, 18].

Gaining weight during pregnancy is ordinary and necessary for the child's growth. Weight gain during this period is usually between 11 and 15 kilos, and the spine must support this body weight. This additional load causes pain in the back. In addition, the weight of the child and the uterus are also growing, and this adds to the pressure on the blood vessels and nerves located in the back and pelvic region [17, 28], which has a higher prevalence of back pain in the third trimester than in the second and first. In our study, it can be due to the same issue, i.e., the more significant increase in the weight of the fetus and mother in the third trimester and the resulting pressure on the mother's back; It has also been mentioned in two studies that the high BMI of the pregnant mother and her excess weight are influential factors for the pregnant woman to suffer from back pain during pregnancy [17, 28].

The exact prevalence of back pain during pregnancy differs in the included studies. Still, their general results are consistent with the finding that pregnant women experience a significant percentage of back pain during pregnancy [4, 18, 31].

A study by Stapleton et al. in Australia on 397 pregnant women reported a prevalence of back pain of 68% [9]. Shijagurumayum Acharya and colleagues also noted in their study on the prevalence of back pain in pregnancy that 34% of pregnant Nepalese women had back pain during pregnancy [13]. In 2021, Shafi et al. conducted a study on 160 pregnant women in Pakistan titled "Prevalence of back pain in pregnancy in women with preeclampsia." Out of 160 people, 55 had back pain during pregnancy. The prevalence of back pain in this study was reported as 68.8% [30]. In 2019, Saxena et al. also conducted a survey titled "Back pain caused by pregnancy in Indian women: prevalence, risk factors and relationship with serum calcium level" on 200 Indian pregnant women with an average age of 9.9 ± 27.1. 80% of pregnancies were reported in these people [7].

In a cross-sectional study conducted by Berber et al. in Turkey in 2020 on 400 pregnant women with an average age of 28.09 ± 5.58, the prevalence of back pain in the third trimester of pregnancy was reported as 45.5% [6]. Rabiee et al. also reported the prevalence of back pain in pregnancy in 63.3% in the first trimester, 63.4% in the second trimester, and 74.2% in the third trimester, which shows the prevalence of back pain during pregnancy is higher in the third trimester [12]. Sencan and colleagues in Turkey reported the prevalence of back pain in pregnancy in the first trimester of pregnancy at 17.43%, in the second trimester of pregnancy at 37.4%, and in the third trimester at 45.86% [32]. Weis et al. also reported the prevalence of back pain in pregnancy at 5.9% in the first trimester, 38% in the second trimester, and 56.1% in the third trimester [31]; According to the results of our study, the above studies also show that the prevalence of back pain during pregnancy is higher in the third trimester.

Back pain during pregnancy is one of the risk factors for back pain after childbirth and can affect different aspects of the sufferers' lives; So, some degrees of movement disorders and insomnia were observed in people suffering from back pain, and the amount of absence from work (in working women) was also reported to be higher [43]. Research shows that in some women, back pain continues in the postpartum period or a new period of back pain begins; the Prevalence of back pain in pregnancy after delivery has been reported in most research to be nearly 40% [9, 13, 31].

In one of the researches, no difference was found in the prevalence of back pain between those who worked inside and outside the home [28]. Still, it was observed that work factors such as heavy work, rotational movements, bending forward and being under pressure in the body position, and back pain after childbirth had an effect [28]. In the study of Hollisaz et al. in 2007 and Ghaderi et al. in 2012, those with a standing job position had a higher probability of back pain [14, 15].

Another study showed a significant relationship between the short height of the mother and back pain during pregnancy [20]. The results of one study showed that long periods of back pain in younger pregnancies cause more prolonged periods of back pain after delivery [16]. Several studies have also investigated the relationship between age and the incidence of back pain during pregnancy, which indicates a direct relationship between the incidence of back pain during pregnancy and increasing age; Back pain is more common among mothers who become pregnant at an older age [7, 14, 20].

Also, those who had a history of back pain in a previous pregnancy were 2.54 times more likely to have back pain in their subsequent pregnancy than those who did not [4, 16]. On the other hand, one of the studies showed that the previous history of the belt, in addition to being a risk factor for back pain after pregnancy, also causes an increase in severity up to six months after delivery [30]. Another study also showed that back pain during pregnancy is not related to back pain before pregnancy but is related to back pain during menstruation and pregnancy [17].

In this study, the prevalence of back pain in the first, second, and third trimesters of pregnancy in Iran was the highest, and one of the reasons for this is that with the increase in the size of the abdomen, pregnant women avoid bending forward to pick up objects [13]. Therefore, they may experience less pain due to a more limited range of motion. This may be the reason for maximum pain in the second trimester and maximum disability in daily activities in the third trimester, also women with more disability often had more pregnancies [13]. While a study also talks about Pregnancy-related transient osteoporosis and finds this factor effective in the effect of pain in the third trimester of pregnancy, Pregnancy-related transient osteoporosis of the hip is a rare condition that manifests with sudden pain located in the groin. region, anterior thigh, and buttocks. It occurs during the third trimester of pregnancy or less frequently during the post-partum period [44, 45].

A study has also reported that the sitting or standing position during the first stage of labor causes an 80% reduction in continuous back pain and a 50% reduction in back pain with contractions. Also, sitting is more comfortable in the late first and second stages of labor [23]. Also, in Mohseni-Bandpei et al.'s study, pregnant women with better general health conditions reported lower back pain intensity [18]. In general, as mentioned, in line with the results of the present study, back pain is a common complication in pregnant mothers, especially in the third trimester of pregnancy. Also, the prevalence of back pain in pregnant women differs in the reviewed studies. This difference may be due to the use of different tools, its dependence on the report of pain, and various factors that play a role in the occurrence of this complication.

## Limitations

One of the limitations of this meta-analysis is that the included studies were limited to research published in English, which means that studies in other languages may have needed to be addressed. In addition, several studies were excluded due to low quality, for example, not reporting prevalence or low sample size.

## Conclusion

According to the results of the present study, the overall prevalence of back pain in pregnancy is 40.5%, which is a significant result and a high prevalence, so the results obtained from the study can be used as the final criteria for proper prevention and treatment planning, and health policymakers can use the results of the present meta-analysis to pay more attention to the complication of back pain during pregnancy, to evaluate this complication and its consequences on the health of pregnant mothers who are part of the active population of the society; Use it as a research priority and implement optimal solutions to prevent and improve this disease.

## Data Availability

Datasets are available through the corresponding author upon reasonable request.

## Abbreviations

RLS: Restless legs syndrome
STROBE: Strengthening the Reporting of Observational studies in Epidemiology
PRISMA: Preferred Reporting Items for Systematic Reviews and Meta-Analysis
